# The mortality-incidence ratio as an indicator of five-year cancer survival in metropolitan Lima

**DOI:** 10.3332/ecancer.2018.799

**Published:** 2018-01-18

**Authors:** Karoline Stenning-Persivale, Maria Jose Savitzky Franco, Alejandra Cordero-Morales, José Cruzado-Burga, Ebert Poquioma, Edgar Díaz Nava, Eduardo Payet

**Affiliations:** 1 School of Medicine, Peruvian University of Applied Sciences, 15067 Lima, Peru; 2 National Cancer Institute, 15038 Lima, Peru

**Keywords:** Peru, mortality, incidence, cancer, survival, cancer registries, mortality/incidence ratio

## Abstract

**Introduction:**

The Mortality–Incidence Ratio complement [1 – MIR] is an indicator validated in various populations to estimate five-year cancer survival, but its validity remains unreported in Peru. This study aims to determine if the MIR correlates directly with five-year survival in patients diagnosed with the ten most common types of cancer in metropolitan Lima.

**Materials and methods:**

The Metropolitan Lima Cancer Registry (RCLM in Spanish) for 2004–2005 was used to determine the number of new cases and the number of deaths of the following cancers: breast, stomach, prostate, thyroid, lung, colon, cervical, and liver cancers, as well as non-Hodgkin’s lymphoma and leukaemia. To determine the five-year survival, the five-year vital status of cases recorded was verified in the National Registry of Identification and Civil Status (RENIEC in Spanish). A linear regression model was used to assess the correlation between [1 – MIR] and total observed five-year survival for the selected cancers.

**Results:**

Observed and estimated five-year survival determined by [1 – MIR] for each neoplasia were thyroid (66.7%, 86.7%), breast (69.6%; 68%), prostate (64.3%, 63.8%) and cervical (50.1%, 58.5%), respectively. Pearson’s *r* coefficient for the correlation between [MIR – 1] and observed survival was = 0.9839. Using the coefficient of determination, it was found that [1 – MIR] (X) captures the 96.82% of observed survival (Y).

**Conclusion:**

The Mortality–Incidence Ratio complement [1 – MIR] is an appropriate tool for approximating observed five-year survival for the ten types of cancers studied. This study demonstrates the validity of this model for predicting five-year survival in cancer patients in metropolitan Lima.

## Introduction

Cancer is one of the most devastating diseases and is among the leading causes of morbidity and mortality worldwide. The World Health Organization reported that 8.2 million people worldwide died of cancer in 2012 [1]. Cancer is a leading public health problem in Peru and also the second leading cause of death in both sexes [2]. Some cancers are preventable and some are potentially curable. It is thus crucial to study indicators such as survival that help us assess how cancers progress and how health systems respond to them [3, 4]. Studying key indicators allows us to measure their effectiveness and profitability, and helps us plan and manage resources for cancer control [6, 7].

Accurate population-based cancer registries are needed in order to estimate general cancer survival across populations and survival for specific types of cancer. The lack of active monitoring, even in developed countries with population-based cancer registries, hinders efforts to make reliable five-year survival estimates. Recent studies have therefore assessed the validity of the mortality-incidence ratio complement [1 – MIR] as an indicator for estimating cancer survival. The MIR complement results from subtracting from the result obtained by dividing the number of deaths by the number of new cases of a specific cancer type recorded during the same time period in a specific population [4, 9, 10]. A higher result of the complement of MIR indicates a better survival rate. A 2010 analysis of seven cancer registries in developing countries examined information on 32 types of cancer, finding that for at least five of the seven countries the complement [1 – MIR] was a good predictor for five-year cancer survival, and largely free of bias [3]. However, the authors also found that the complement overestimated survival in some cancers (oral and liver cancers) and underestimated survival in other cancers (soft tissue, bone, breast, prostate, and oesophagus cancers, as well as myeloma and leukaemia).

To our knowledge, no study has been developed using data from population-based cancer registries in developing countries to estimate the survival. Thus, the validity of the MIR complement as an indicator of survival in these countries remains untested. In Peru, the Metropolitan Lima Cancer Registry (RCLM) is the only registry subject to a quality control system that scrutinises the data that it collects and processes. It has an international certificate and has been an active member of the International Association of Cancer Registries since its creation. This registry was created in 1990, and collects information on all new cancer cases diagnosed in all public and private institutions in Lima and Callao Provinces. The RCLM covers date from 2004 to 2005, when 31,226 new cancer cases were recorded in Lima and Callao [8]. Conducting survival studies using registries such as the RCLM is challenging due to problems related to active monitoring of cancer patients. Survival studies also pose funding and logistical challenges in developing countries such as Peru.

This study aims to assess the validity of the MIR as a predictor of cancer patient survival in metropolitan Lima.

## Methodology

### Source material

The data used in this study was obtained from RCLM. This information included cancer cases recorded by the National Cancer Institute (INEN) from 2004 to 2005 (the most recent RCLM volume at the time of the study) [8]. This study population includes patients residing in metropolitan Lima (Lima and Callao) diagnosed with some type of cancer (identified by pathology), during the study period (2004–2005), as well as patients who died of cancer in the same period of time. Metropolitan Lima includes 49 districts: 43 within Lima Province and 6 within Callao Constitutional Province. ‘Incidence data are collected from all healthcare institutions in the registry area: public hospitals, the social health insurance system, institutions serving the armed forces and police, private clinics, pathology and haematology laboratories, and private medical practices’ [8]. Mortality data was collected by checking the five-year vitality status in the National Registry of Identification and Civil Status (RENIEC) of patients diagnosed in 2004–2005.

Data on new cases, mortality, and the MIR was acquired from the RCLM from 2004–2005. A new case was considered as a person diagnosed with a cancer (by pathology) residing in metropolitan Lima during the years 2004–2005. Cancer deaths included deaths that occurred during the same period and whose cause of death was one of the previously mentioned cancers. Regarding the observed survival data, it was estimated using a study sample of the new cases recorded in the RCLM and the vitality status, of each new case of the sample, was verified five years from the date of diagnosis using data from the RENIEC. RENIEC contains all the data from the national identity document emitted to all Peruvians (born or nationalised) with the unique identity number, names and surnames, date of birth, sex, marital status, address, and the status of the person. Authorised staff from the INEN Department of Epidemiology and Cancer Statistics reviewed this data under an existing institutional agreement.

### Sample and sampling

#### Procedure for calculating survival study sample size

Ten cancer types were taken for the study, but it was found that the number of new cases of each one varied significantly; therefore, a representative sample size was calculated for the seven cancer types that exceed 1,000 reported cases (simple random selection without replacement, calculated using the formula *K* = *N*/*n*). Those seven cancer types were breast, stomach, prostate, lung, colon, and cervical cancers and non-Hodgkin’s lymphoma. For the cancers with the number of cases reported below 1,000 (liver and thyroid cancer and leukaemia) all cases reported were included. At the same time, subjects who were diagnosed with cancer from death certificates were excluded from the analysis, as including them would impair survival time calculation [11].

The sample size required was calculated for each cancer type using a simple random sampling without replacement, with the following formula determining required sample size:

$$n=\frac{N\times P\times Q}{(N-1\mathrm{)(}\frac{E}{Z}{)}^{2}+P\times Q}$$

where *n* is the sample size; *N* is the patient population with each type of cancer; *P* is the proportion of the population at risk for each type of cancer; *Q* is the complement of *P* (*Q* = 1 – *P*); *E* is the estimated error *E* = 0.035 (3.5%); and *Z* is 95% confidence (1.96).

Furthermore, sample size was increased 10% to account for the risk of not finding all selected cases (Table 1).

#### Procedure for selecting the cases used in the sample

The cases were selected systematically and only for cancers that required calculating the sample size. A sampling frame was created for each type of cancer, which included all the sampling units that form our population, then these units were organised according to reference hospital, numbering them from 1 to *N*.

Our selection interval was determined by using the below formula:

$$K=\frac{N}{n}$$

where *N* is the population size and *n* is the sample size.

For all cancers, a non-integer value ‘*K*’ was obtained, rounded to the nearest whole number by selecting a random number ‘*a*’ between 1 and *N*, adding the *K* constant to select cases for the sample.

### Statistical analysis

#### Phase 1: MIR calculation

The numbers of deaths and new reported cases were obtained for each type of cancer studied from the RCLM. This allowed us to determine the MIR.

$$\mathrm{MIR}=\frac{\mathrm{number}\;\mathrm{of}\;\mathrm{deaths}}{\mathrm{number}\;\mathrm{of}\;\mathrm{new}\;\mathrm{cases}}$$

With these data, the complement [1 – MIR] was calculated, expressed as a percentage between 0 and 100. Values approaching 0% represent a poor survival rate and those approaching 100% represent an excellent survival rate.

#### Phase 2: Observed survival analysis

**Figure 1.**
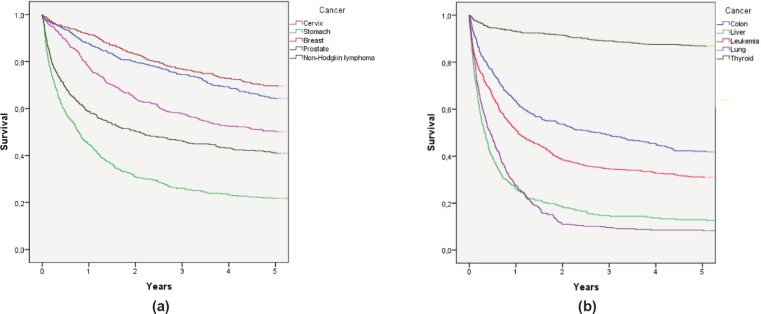
(a) Observed survival curves for the five most frequent cancers and (b) observed survival curves for the five remaining cancers.

To determine survival, the Kaplan–Meier method was used. Alive cases were considered when the last check-up occurred at least five years after diagnosis, and deceased cases were considered when RENIEC showed a date of death within five years of diagnosis. The IBM SPSS Statistics 22 program was used to determine the five-year survival probability for each cancer [see Figure 1 (a) and (b)].

#### Phase 3: Analysis of correlation between [1 – MIR] and observed five-year survival in the sample

To measure the correlation between [1 – MIR] and observed five-year survival, Pearson’s r coefficient was used. For this, the variable [1 – MIR] was considered as (*X*) and observed survival as (*Y*). A correlation coefficient of 0.7–1 was perceived as strong, and a coefficient between 0.2 and 0.4 as weak.

**Figure 2.**
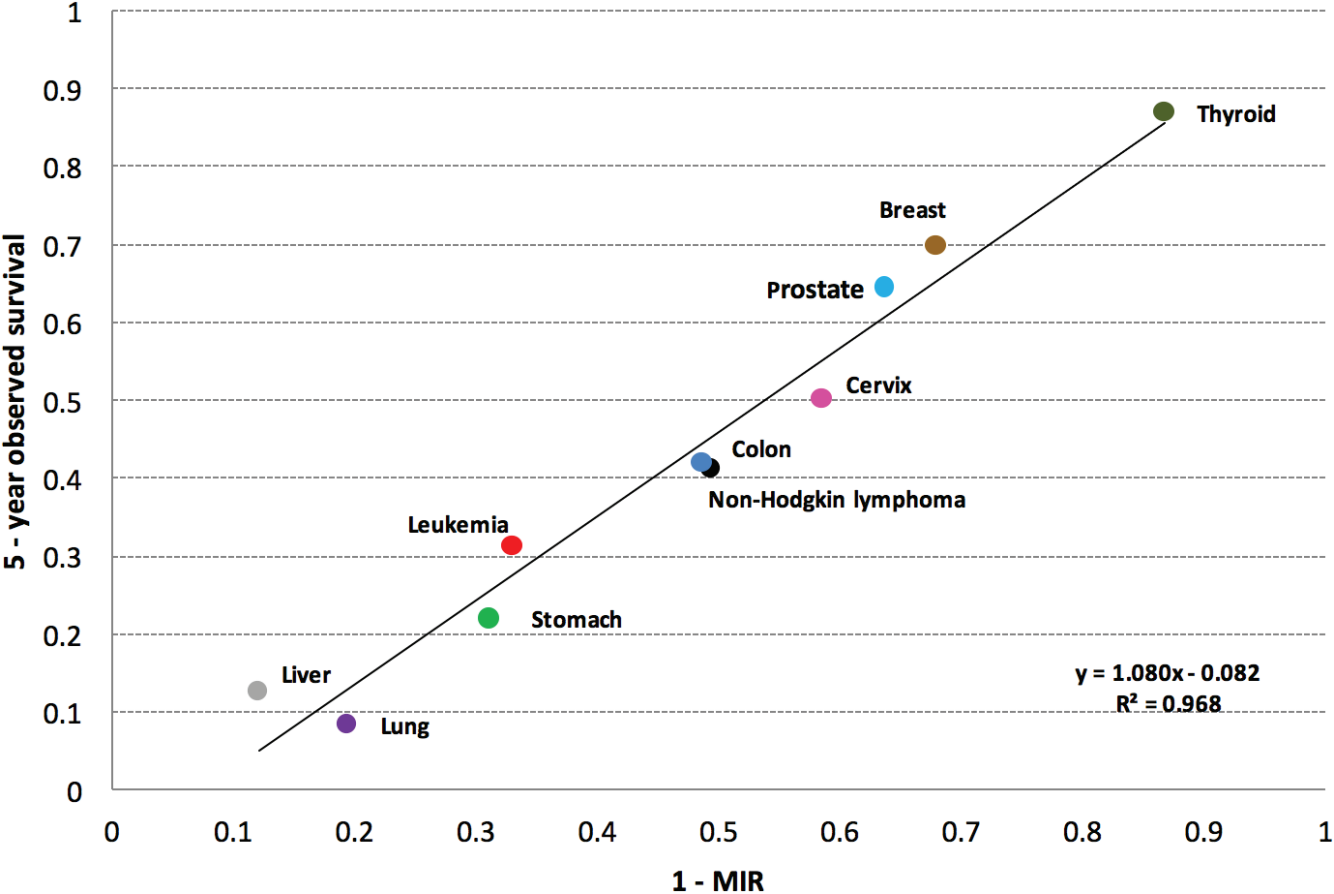
Regression line of five-year observed survival on 1 – MIR.

By corroborating a strong correlation between the variables [1 – MIR] and observed survival with Pearson’s r coefficient, then, to measure and express this relationship using a function or mathematical model, a simple linear regression model (using the ten types of cancer as a whole) was used to explain the behaviour of observed survival as a function of [1 – MIR]. Using the data obtained, a linear regression model was created with the following equation: *Y* = *a* + *b X* (see Figure 2).

### Ethics

The Ethics Committees of the Peruvian University of Applied Sciences and the National Cancer Institute (INEN) approved this study. Permission to access data under an agreement between RENIEC and INEN was obtained, and a coding system was used to maintain the confidentiality of each patient.

## Results

The values for the MIR and its complement [1 – MIR], as well as observed five-year survival values were obtained (Table 2). These values show that the cancers with the highest percentage of estimated five-year survival [1 – MIR] are thyroid (86.7%), breast (68%), prostate (63.8%), and cervical cancer (58.5%). Similarly, the cancers with lower estimated survival percentages are lung (19.4%), liver (12.2%), and stomach (31.1%) cancers and leukaemia (33.2%). These results were obtained using the MIR complement calculated from data in the Metropolitan Lima Cancer Registry.

These findings were consistent with what was obtained with the observed survival from the database created using RENIEC data. These results appear in Figure 1(a) and 1(b), where each curve represents a type of cancer and observed survival time (*X*-axis). These values show that the cancers with the highest percentage of observed five-year survival are thyroid (86.7%), breast (69.6%), prostate (64.3%), and cervical cancer (50.1%). Similarly, the cancers with a lower percentage of observed survival are lung (8.2%), liver (12.6%), and stomach (21.8%) cancers, and leukaemia (31.0%). These percentages are similar to the survival results obtained [1 – MIR]; however, in the cases of stomach and lung cancer, it was observed that the observer survival underestimated the estimated five-year survival [1 – MIR].

A value of *r* = 0.9839 was determined to show the correlation between [1 – MIR] (*X*) and observed survival (*Y*). Using this data, the following linear regression model *Y* = 1.0807 *X* = 0.082 was obtained (Figure 2). Using this information, a coefficient of determination, R^2^ = 96.82%, was found. This indicates that [1 – MIR] (*X*) captures 96.82% of observed survival (*Y*).

Figure 2 shows the various types of cancers were those with a better approximation to the observed survival using [1 – MIR] were breast (1.7%), prostate (0.5%), liver (0.4%), and thyroid (0.0%) cancers, and leukaemia (2.2%). The values reported are the percentage differentials between five-year observed survival and the complement [1 – MIR]. The remaining cancers also did not show a significant difference, but the five listed above showed the lowest differences of all the cancers studied.

## Discussion

Multiple studies in different countries have used the MIR as an indicator of five-year survival for various types of cancer. Parkin *et al* confirmed that the MIR is a good indicator of five-year survival when collection of neoplasia incidence and mortality data is rigorous and consistent [10]. To our knowledge, however, no one has studied the MIR as a tool for evaluating survival in Peru. Cancer incidence and mortality vary based on lifestyle, genetics, environment, access to a healthcare system and treatment, and other factors. There was then a need to assess whether MIR is a valid tool for the population of metropolitan Lima.

The MIR is a public health tool that makes estimating survival for each type of neoplasia more feasible. Studies of survival allow us to understand patterns and establish the behaviour and burden of each type of neoplasia. The MIR helps us predict future burdens and trends, allowing us to project incidence and mortality for each neoplasia and monitor the disease. These steps are essential to oncology [4, 13]. The MIR also allows us to retrospectively assess populations with shorter or longer five-year survival. This information can then be connected to cancer prevention, diagnosis, and treatment methods in use during the same time period and at the same time determine what actions were taken at the time, and focus on making decisions that will achieve better survival in the future [12, 14].

The results of our study indicated that the observed survival findings were consistent with the estimate made using the MIR complement. By evaluating [1 – MIR] and observed survival in the linear regression model, it was found that the ten types of cancer studied fall in a nearly perfect line *y* = *x* on the regression line, suggesting a strong correlation. Obtaining a coefficient of determination of *R*^2^ = 0.9682, it shows that the variations in [1 – MIR] (*X*) explain 96.82% of the variation in observed survival (*Y*). It is indicated that the MIR is a valid indicator for five-year survival for the types of cancers studied in the population of metropolitan Lima.

Of the ten cancers studied, nine had a difference of less than 10% between the observed survival and the survival estimated by the 1–MIR. Within these cancers, we must emphasise the thyroid cancer whose observed survival was the same as that estimated by the 1-MIR (86.75% for both survivals). On the other hand, only one cancer (lung cancer) presented an overestimation of more than 10%, with observed survival of 8.2% versus survival estimated with 1–MIR of 19.4%. In general, there was a tendency to overestimate, but not more than 10% for the great majority of cancers.

Although our results confirm the use of MIR to evaluate five-year survival, the limitations of this study must be recognised. First, the MIR can only serve as a valid measure with a population-based cancer registry. Maintaining such registries can be as complicated as actively monitoring cases in order to achieve accurate estimates of survival. In Peru, the only population-based cancer registry is the RCLM, which collects information from all public and private hospitals in the Lima metropolitan area [8]. The efforts of the INEN to maintain the RCLM are complex and require staff, logistics, and a budget. These challenges have prevented INEN from creating cancer registries in other cities and from analysing RCLM data from other years. This lack of resources is a limitation. MIR-based survival calculations require INEN or another institution to continue collecting data and maintain population-based cancer registries over the years. However, this reality is not the same for all countries, other nations like Brazil for example, have achieved greater success in these areas. Brazil has up-to-date registries containing significant data that allows public health agencies to actively monitor patients over time [7].

Another limitation resulted from gaps in the RENIEC data that made it difficult to confirm survival of selected cases. A portion of cases lacked current information and this could generate a sub-registry of cases from our sample [11]. Therefore, we decided to increase the sample size during the study. Additionally, another point to mention is the simple random sampling used, not stratifying by sex, age, stage of diagnosis, or any other co-variable due to lack of time and funds. However, the aim of our study was to analyse the population of metropolitan Lima as a unit, assessing general survival for each type of neoplasia. Therefore, it should be considered that not stratifying the sample may represent a limitation.

At the same time, another limitation was not calculating MIR for all cancer types, but only for ten specific ones. Ten cancers can be a small number for a linear regression model. Nonetheless, starting with these representative types of cancers is a beginning or starting point to complement them with more areas of studies. Also, it must be emphasised that the studied cancers were selected as most representative in the study population (choosing those with the highest and lowest mortality and the highest incidence, affecting both sexes).

## Conclusion

Finally, it can be established that the differences between MIR-estimated survival and observed survival are insignificant. The MIR thus appears to be an appropriate tool for predicting five-year survival for the types of cancer studied in metropolitan Lima. This study then confirms the validity of the MIR only for this population, but provides the basis for new studies and uses of the MIR. Oncologists, epidemiologists, public health researchers, and healthcare managers can use the MIR to study the population of metropolitan Lima [13, 15].

## Conflicts of interest

The authors declare that they have no conflicts of interest.

## Figures and Tables

**Table 1.** Ten cancers used in the study with their sample size.

Cancer	New Cases*	Sample Size	Final Sample Size**
Breast	2 749	548	603
Stomach	2 235	517	569
Prostate	2 342	554	610
Cervix	1 602	517	569
Lung	1 255	353	389
Non-Hodgkin Lymphoma	1 423	506	557
Colon	1 047	449	494
Leukemia	746	-	-
Thyroid	743	-	-
Liver	301	-	-

* New cases: New cases minus those cases diagnosed by death certificate.

** Final sample = Sample size + 10%

- No sample size applied (Sample size was not calculated; all cases were used)

**Table 2.** Results of the Complement of the Mortality-Incidence Ratio (1-MIR) and the 5-year observed survival of the ten cancers used.

Cancer	Deaths	New Cases	MIR	Survival (1- MIR) (X)	5-year Observed Survival (Y)
Breast	925	2886	32.0%	68.0%	69.6%
Stomach	1950	2831	68.9%	31.1%	21.8%
Prostate	993	2744	36.2%	63.8%	64.3%
Cervix	687	1656	41.5%	58.5%	50.1%
Lung	1366	1695	80.6%	19.4%	8.2%
Non-Hodgkin Lymphoma	776	1533	50.6%	49.4%	41.0%
Colon	638	1248	51.1%	48.9%	41.8%
Leukemia	625	935	66.8%	33.2%	31.0%
Thyroid	101	760	13.3%	86.7%	86.7%
Liver	576	656	87.8%	12.2%	12.6%
